# Midregional Proadrenomedullin (MRproADM) Serum Levels in Critically Ill Patients Are Associated with Short-Term and Overall Mortality during a Two-Year Follow-Up

**DOI:** 10.1155/2020/7184803

**Published:** 2020-08-07

**Authors:** Lukas Buendgens, Eray Yagmur, Axel Ginsberg, Ralf Weiskirchen, Theresa Wirtz, Samira Abu Jhaisha, Albrecht Eisert, Tom Luedde, Christian Trautwein, Frank Tacke, Alexander Koch

**Affiliations:** ^1^Department of Medicine III, RWTH-University Hospital Aachen, Germany; ^2^Medical Care Center, Dr Stein and Colleagues, Mönchengladbach, Germany; ^3^Institute of Molecular Pathobiochemistry, Experimental Gene Therapy and Clinical Chemistry, RWTH-University Hospital Aachen, Germany; ^4^Hospital Pharmacy, RWTH-University Hospital Aachen, Germany; ^5^Institute of Clinical Pharmacology, RWTH-University Hospital Aachen, Germany; ^6^Department of Hepatology and Gastroenterology, Charité University Medicine Berlin, Germany

## Abstract

Adrenomedullin (ADM) is a peptide with pleiotropic effects in systemic inflammation. Its more stable precursor protein midregional proadrenomedullin (MRproADM) can be measured more reliably compared to ADM. Our objective was to investigate the potential role of MRproADM as a diagnostic and prognostic biomarker in critically ill patients at the intensive care unit (ICU). We therefore measured MRproADM in 203 ICU patients and 66 healthy controls. We found that MRproADM levels are significantly increased in critically ill patients as compared to healthy controls. MRproADM levels are significantly increased in patients with sepsis, but its diagnostic value for identifying sepsis is numerically lower than that of established markers (e.g., interleukin-6, C-reactive protein, and procalcitonin). MRproADM levels are closely correlated to endothelial and organ dysfunction, inflammation, and established clinical scores (APACHE II, SOFA, and SAPS2). MRproADM concentrations correlate with vasopressor use but not fluid balance. Increased MRproADM levels (cut − off > 1.4 nmol/L) in critically ill patients are independent predictors of ICU and overall mortality during a follow-up of up to 26 months (OR 3.15 for ICU mortality, 95% CI 1.08-9.20, *p* = 0.036; OR for overall mortality 2.4, 95% CI 1.12-5.34, *p* = 0.026). Our study demonstrates the potential of MRproADM serum levels as a prognostic biomarker in critical illness for ICU mortality and long-term survival during follow-up.

## 1. Introduction

Adrenomedullin (ADM) is a 52-amino-acid peptide first discovered in 1993 in pheochromocytoma cells [1]. It is expressed in various tissues (e.g., adrenal medulla, pancreas, heart, aorta, kidneys, lungs, and macrophages) [2] and has pleiotropic biological effects, including vasodilation, inotropy, immune modulation, and diuretic effects, especially in the presence of proinflammatory stimuli [3, 4]. The vasodilatory properties of ADM are of special interest in the pathophysiology of sepsis. ADM has direct effects by stimulating receptors on smooth vascular muscle cells and consecutively increases intracellular cAMP [5] and indirect effects by increasing the activity of inducible NO synthase (iNOS) [6] augmenting effects of interleukin 1 (IL1) [7]. Consistent with this, it has been suggested that ADM exerts an important role in the hyperdynamic phase of sepsis and the transition to the subsequent hypodynamic phase [8].

ADM itself underlies rapid clearance and is difficult to measure in clinical settings. A nonfunctional fragment of its precursor protein, midregional fragment of proadrenomedullin (MRproADM) is larger and more stable than active ADM [9]. Thus, MRproADM measurements can be used as reliable surrogates in predicting biological effects of ADM [10].

Previous studies showed the prognostic value of elevated MRproADM serum levels in patients with pneumonia [11–13], myocardial infarction [10], or patients in the emergency department [14, 15] as well as critical illness and sepsis [16–21]. Nevertheless, the potential of MRproADM as a biomarker in the ICU setting has not been satisfyingly elucidated. Therefore, we conducted a large prospective clinical study focusing on the association between MRproADM and organ dysfunction in critical illness as well as its role as a prognostic tool for ICU and long-term mortality.

## 2. Materials and Methods

### 2.1. Study Design

Written informed consent was obtained from the patient, his or her spouse, or the appointed legal guardian. Excluded were patients with expected short–term (<72 h) intensive care treatment (e.g., postinterventional observation) [22]. Follow-up data was collected by directly contacting the patient, the patients' relatives, or their primary care physician. The Third International Consensus Definition for Sepsis and Septic Shock was used as a post hoc definition for discriminating sepsis and nonsepsis patients [23]. To evaluate organ dysfunction, a panel of serum markers such as cystatin C, albumin, pseudocholinesterase, prothrombin time, or N-terminal pro-B-type natriuretic peptide was used. Moreover, we used composite parameters such as the Horovitz index (P_a_O2/F_i_O_2_ ratio) as a marker of pulmonary dysfunction and established scores such as the acute physiology and chronic health evaluation II (APACHE II) score, the sequential organ failure assessment (SOFA) score, and the simplified acute physiology score 2 (SAPS2) as surrogates for overall disease severity. The Charlson comorbidity index [24] was used to classify comorbidities.

As a control cohort, we recruited healthy blood donors with normal values for blood counts, C-reactive protein, and liver enzymes. Our study protocol was accepted by the local ethics committee (ethics committee of the University Hospital Aachen, RWTH-University, Aachen, Germany, reference number EK 150/06) and conducted in agreement with the 1964 Declaration of Helsinki.

### 2.2. MRproADM Measurements

Plasma MRproADM concentration (epitopes covering amino acids 68-94, equivalent to MRproADM and active ADM) was determined using an automated immunofluorescent assay based on TRACE technology (time-resolved amplified cryptate emission technology assay), according to the manufacturer's instructions (B.R.A.H.M.S MRproADM Kryptor, #829.050, BRAHMS AG, Henningsdorf, Germany) [25]. EDTA-blood samples were collected at the time of admission (before specific therapeutic measures). After centrifugation at 4°C for 10 minutes, plasma aliquots of 1 ml were frozen immediately at -80°C. The assay has analytical detection limit of 0.05 nmol/L. The person performing the measurements was fully blinded to any clinical or other laboratory data of patients or controls.

### 2.3. Statistical Analysis

Due to the skewed distribution of most of the parameters, distributions of data are given as median and range. To assess significant differences, the Mann–Whitney *U* test, chi-squared test, or Fisher's exact test were used. Correlations between variables were analysed using the Spearman correlation tests. Multivariate logistic regression analysis was used to investigate independence of MRproADM as a prognostic marker for mortality. Here, only parameters with no or low correlation were implemented to avoid multicollinearity [26]. The Kaplan Meier method was used to plot survival curves, with the Log-Rank Test for significance. Receiver operating characteristic (ROC) curve analyses were used to assess diagnostic accuracy of markers or scores. The DeLong method was used to compare AUCs [27]. The Youden index (the sum of sensitivity and specificity minus one) was used to calculate optimum cut-offs [28]. Statistical analyses were carried out with SPSS version 23 (SPSS, Chicago, IL, USA), and MedCalc version 19 was used for DeLong testing (MedCalc Software, Ostend, Belgium).

## 3. Results

### 3.1. MRproADM Serum Levels Are Increased in Critically Ill Patients and Associated with Sepsis

To investigate the role of MRproADM as a relevant biomarker in a nonselected cohort of medical critically ill patients, we measured MRproADM serum levels in 203 patients at the time of admission to our medical ICU prior to therapeutic interventions.

Measurements of MRproADM in healthy controls (*n* = 66) were all below the analytical detection limit of 0.05 nmol/L.

**Figure 1:**
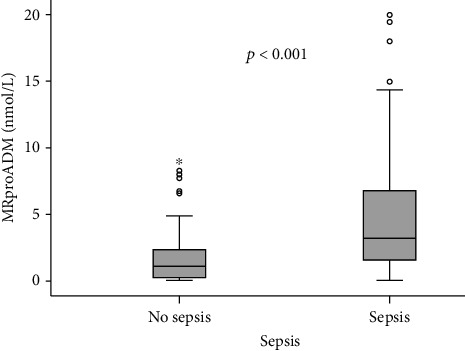
Serum MRproADM concentrations in critically ill patients and sepsis. MRproADM levels were significantly higher in patients with sepsis compared to ICU patients without sepsis (median 3.2 vs 1.1 nmol/L; *p* < 0.001).

In 136 patients, sepsis and septic shock were the major causes of ICU admission. Infection sites were the lung (*n* = 71), abdomen (*n* = 26), urogenital tract (*n* = 8), and other foci (*n* = 31). Nonseptic ICU patients were admitted due to cardiopulmonary diseases (*n* = 28), pancreatitis (*n* = 9), decompensated liver cirrhosis (*n* = 8), and other nonseptic diseases (*n* = 22) (Table 1). Compared to nonsepsis patients, MRproADM serum concentrations were significantly increased in patients with sepsis (median 3.2 vs 1.1 nmol/L; *p* < 0.001; Figure 1, Table 2). Both, sepsis and nonsepsis patients did not differ in age or sex. Nevertheless, sepsis patients had significantly higher APACHE II scores and increased need for vasopressors (Table 2).

In order to analyse the diagnostic potential of MRproADM for identification of sepsis, we performed ROC analyses comparing MRproADM with clinically established and routinely used markers such as procalcitonin (PCT) and C-reactive protein (CRP). PCT and CRP showed AUCs of 0.767 (95% CI 0.69-0.845) and 0.840 (95% CI 0.773-0.907), respectively. MRproADM displayed a numerically lower performance in diagnosing sepsis with an AUC of 0.731 (95% CI 0.647-0.814). There was no significant difference between either pair of markers (using the DeLong test).

Next, we tested if a combination of MRproADM with established and routinely used markers such as CRP, PCT, and IL6 could increase the diagnostic accuracy for sepsis using a composite score. Here, the range of each biomarker was divided into quartiles and one score point was given for each quartile of the biomarkers value. Interestingly, not any combination of MRproADM with CRP, PCT, and/or IL6 could increase the diagnostic accuracy for sepsis (data not shown). Additionally, we did not observe significant differences for MRproADM levels in infections with either gram-positive or gram-negative bacteria (data not shown).

### 3.2. MRproADM Levels in Critically Ill Patients Correlate with Clinical Scores, Organ Dysfunction, and Obesity

**Figure 2:**
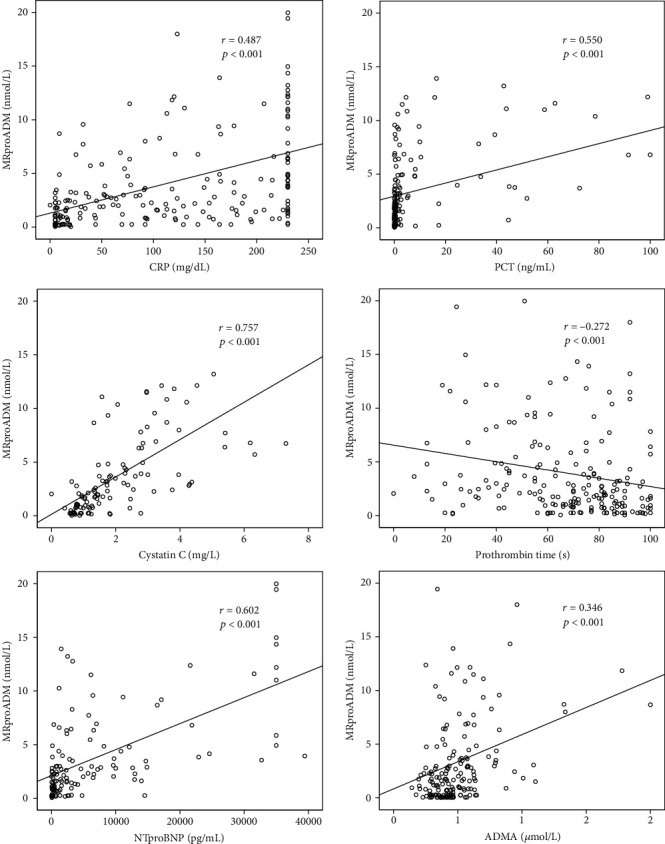
Serum MRproADM levels in critically ill patients correlate with inflammation and organ failure. Correlation analyses revealed associations between serum MRproADM and biomarkers of systemic inflammation (e.g., CRP), renal failure (e.g., cystatin), hepatic dysfunction (e.g., prothrombin time), cardiac failure (e.g., NT-proBNP), or endothelial dysregulation (e.g., ADMA).

We performed extensive correlation analyses to evaluate potential associations between MRproADM and biomarkers of organ dysfunction and clinical scores (Table 3, Figure 2). We found significant correlations between MRproADM serum concentrations and markers of renal dysfunction (e.g., cystatin C, *r* = 0.757, *p* < 0.001), reduced hepatic function (e.g., albumin *r* = −0.456, *p* < 0.001; pseudocholinesterase *r* = −0.499, *p* < 0.001; and prothrombin time *r* = −0.272, *p* < 0.001), and heart failure (e.g., N-terminal pro-B-type natriuretic peptide (NT-proBNP), *r* = 0.602, *p* < 0.001). The Horovitz index (P_a_O2/F_i_O_2_ ratio) as a marker of pulmonary dysfunction showed no significant correlation. Furthermore, MRproADM serum concentrations showed correlations with markers of systemic inflammation (e.g., CRP, *r* = 0.487, *p* < 0.001; interleukin 6 (IL6), *r* = 0.301, *p* < 0.001; and TNF*α*, *r* = 0.576, *p* < 0.001). As MRproADM has been described as a biomarker of endothelial dysfunction, we performed correlation analyses with other endothelium-derived markers such as symmetric dimethylarginine (SDMA) and asymmetric dimethylarginine (ADMA) as well as C-terminal proendothelin-1 (CTproET1) [29–31]. In fact, these biomarkers are closely correlated to MRproADM serum concentrations (SDMA, *r* = 0.616, *p* < 0.001; ADMA, *r* = 0.346, *p* < 0.001; and CTproET1, *r* = 0.870, *p* < 0.001). Consistent with the association to organ failure and endothelial dysfunction, MRproADM levels were found to strongly correlate with administered vasopressor doses (*r* = 0.222, *p* = 0.002), which were routinely used to maintain a mean arterial blood pressure > 65 mmHg. However, we could not demonstrate a correlation between MRproADM serum concentrations and fluid balance at day 1, 3, or 5, respectively (detailed data not shown).

Additionally, elevated MRproADM levels were associated with disease severity as displayed by highly significant correlations with the APACHE II score (*r* = 0.333, *p* < 0.001), SOFA score (*r* = 0.266, *p* < 0.001), or SAPS2 score (*r* = 0.460, *p* < 0.001; Table 3). Interestingly, MRproADM was increased in obese patients with a body mass index (BMI) > 30 kg/m^2^ as compared to patients with a BMI ≤ 30 kg/m^2^ (median 1.9 nmol/L vs 3.0 nmol/L, *p* = 0.039). Preexisting diabetes mellitus was not associated with MRproADM levels.

### 3.3. MRproADM Levels at Admission to the ICU Are Independent Predictors of ICU and Overall Mortality

The correlation of MRproADM levels with clinical scores of disease severity and biomarkers of organ dysfunction prompted us to investigate the value of MRproADM in predicting mortality in ICU patients.

During ICU treatment, 21.2% of all patients died. Overall mortality was 38.9% during the whole observation period of up to 26 months. Patients with fatal ICU outcome showed significantly higher serum concentrations of MRproADM than ICU survivors (median 3.6 vs 2.2 nmol/L; *p* = 0.017, Table 4).

We used ROC for analysing the prognostic value of MRproADM in predicting ICU and overall mortality. For ICU mortality, we could demonstrate that the AUC of MRproADM (0.670; 95% CI 0.556-0.785) is lower than the widely used APACHE II score (0.701; 95% CI 0.577-0.825) and higher than procalcitonin (0.574; 95% CI 0.463-0.686). However, there was no significant difference between either pair of markers (using the DeLong test).

**Figure 3:**
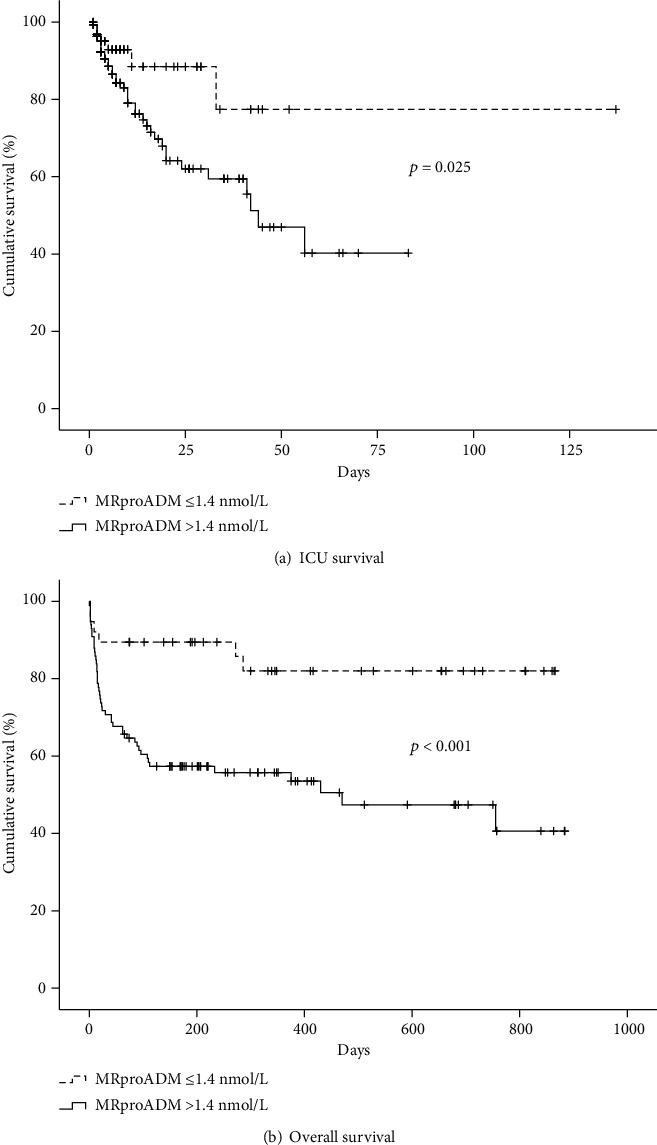
Prediction of ICU mortality by MRproADM serum levels. (a) Kaplan-Meier survival curves of ICU patients are displayed, showing that patients with MRproADM serum levels below a cut-off value of 1.4 nmol/L showed better outcome at the ICU (*p* = 0.025, Log-Rank-test). (b) Kaplan-Meier survival curves of ICU patients are displayed, showing that patients with MRproADM serum levels below a cut-off value of 1.4 nmol/L showed better outcome during follow-up (*p* < 0.001, Log-Rank-test).

Utilizing the Youden index based on sensitivity and specificity from the ROC analysis, a MRproADM cut-off of 1.4 nmol/L performed best in predicting ICU mortality and was used for further analyses. In fact, the strong association of ICU mortality and MRproADM levels higher than 1.4 nmol/L was confirmed in a Kaplan-Meier survival curve analysis (Figure 3(a)). Given these findings, we further investigated if MRproADM might predict overall survival. Indeed, overall survivors showed significantly lower values of MRproADM than nonsurvivors at admission to the ICU (2.0 nmol/L vs 3.2 nmol/L; *p* = 0.006). For overall mortality, MRproADM showed no significant difference in ROC analysis (AUC 0.655; 95% CI 0.557-0.753) as established scores (e.g., APACHE II; AUC 0.616; 95% CI 0.516-0.717) or biomarkers (e.g., PCT; AUC 0.586; 0.486-0.687). We next tested if MRproADM improves the prognostic performance of APACHE II, SOFA, and SAPS2 when used as a composite model. We therefore divided the range of each parameter into quartiles, and one score point was given for each quartile of the score parameter. In fact, the combination of APACHE II and MRproADM improved the prognostic value for overall mortality minimally but significantly (AUC 0.616 vs 0.658, *p* = 0.0479). The combination of MRproADM with either SAPS2 or SOFA had no impact on the prognostic value (detailed data not shown).

In line with our findings for ICU mortality, Kaplan-Meier survival curve analysis for overall mortality showed excellent discrimination between overall survivors and nonsurvivors using the same cut-off of 1.4 nmol/L (Figure 3(b)).

Given the close correlation of MRproADM with biomarkers of organ failure and inflammation, we conducted multivariate logistic regression to determine independence as a prognostic marker. We included age, markers of inflammation (CRP), renal (cystatin C), circulatory (i.e., lactate), hepatic dysfunction (prothrombin time), and overall disease severity displayed by the APACHE II score in our analyses. MRproADM, age, APACHE II, and lactate showed significance in univariate analysis and were included in the multivariate testing. Here, we could demonstrate that high MRproADM levels were independent predictors of ICU and overall mortality in critically ill patients (OR 3.15 for ICU mortality, 95% CI 1.08-9.20, *p* = 0.036; OR for overall mortality 2.4, 95% CI 1.12-5.34, *p* = 0.026, Table 5). Sensitivity, specificity, negative predictive value, positive predictive value, and likelihood ratios for the chosen cut-off of 1.4 nmol/L are shown in Table 6.

## 4. Discussion

MRproADM has been investigated as a biomarker for diagnosis and prognosis in sepsis and has been further linked to organ failure. Nevertheless, the potential of MRproADM as a biomarker in the ICU setting has remained blurry. This prompted us to conduct this study to explore the role of MRproADM in a large cohort of critically ill patients who we followed over a period of up to 26 months.

We could demonstrate that MRproADM is increased in critically ill patients compared to healthy controls with the highest values found in sepsis patients. MRproADM closely correlated with markers of systemic inflammation, such as CRP, IL6, or TNF*α*. Moreover, we observed highly significant associations with biomarkers of endothelial dysfunction (SDMA, ADMA, and C-CTproET1). Additionally, MRproADM levels were associated with disease severity by correlations to composite clinical scores (APACHE II, SOFA, and SAPS2). Finally, we demonstrated the prognostic value of MRproADM for mortality not only during ICU treatment but also in the long-term follow-up of up to 26 months.

The systemic inflammatory response in sepsis is complex. Therefore, it has been suggested to use a combination of several biomarkers to increase diagnostic effectiveness. In a retrospective study with 104 patients with sepsis, it has been demonstrated that the combination of PCT, MRproADM, and TNF*α* had the best performance in early detection of sepsis compared with single biomarkers [32]. In contrast, in a secondary analysis of a prospective multicenter study, MRproADM did not improve the diagnostic accuracy for sepsis when added to the composite model of clinical parameters [33].

In line with these findings, in our study, the diagnostic accuracy of MRproADM for sepsis was inferior to clinically used biomarkers (CRP, PCT, and IL6) and the combination of MRproADM with any of those markers did not improve diagnostic performance. This discrepancy might be owed to the fact that in the retrospective study, sepsis was defined as the presence of systemic inflammation syndrome (SIRS) and positive blood cultures, whereas in our study, the current Sepsis-3 definition [23] was applied.

In previous studies, MRproADM was proposed as an independent predictor of heart failure and myocardial infarction and as a biomarker for risk stratification in this setting [34]. In our study, we could demonstrate a close link between serum levels of MRproADM and NT-proBNP as an established biomarker of acute and chronic heart failure. Pathophysiologically, ADM exerts potent and long-lasting vasodilatory effects [1]. In line with this, we observed strong correlations between MRproADM and biomarkers reflecting endothelial dysfunction such as ADMA, SDMA, or CTproET1 [29–31]. Prior studies showed increased MRproADM levels in patients who received vasopressor treatment on admission [35]. Moreover, in our study, we demonstrated a dose-dependent correlation between MRproADM levels and vasopressor treatment. In contrast to previous studies [16], we did not observe a significant correlation of serum MRproADM levels with volume demand as expressed by the fluid balance on day 1, 3, or 5 of ICU treatment. Given these findings, one might speculate that MRproADM as a biomarker of decreased vascular tonus could potentially guide circulatory support therapy by indicating a pronounced need of vasopressors rather than volume substitution.

MRproADM levels were not only related to cardiac and endothelial dysfunction but also to impaired renal and hepatic function. Taken together, MRproADM serum levels seem to mirror the extent of organ failure in critical illness, comprising the most prominent organ systems (circulation, liver, heart, and kidney). Thus, it is not surprising that recent large studies found MRproADM to be strongly associated with mortality both in patients in the emergency department [36] and in the intensive care unit [37] and can possibly be used to identify high-risk patients. Fitting in this context, besides known established risk factors such as APACHE II and the biomarker lactate, we identified MRproADM levels as an important and independent predictor of both ICU and overall mortality during follow-up. A remaining question is the optimal MRproADM cut-off for identifying patients at risk. For example, the trial of Saeed et al. [36] found a similar cut-off (1.54 nmol/L) as we did (1.4 nmol/L) while Elke et al. used a considerably higher cut-off (2.75 nmol/L) [37].

Our study had some shortcomings. These include the lack of longitudinal measurements of MRproADM, which could further elucidate its role in prognostic assessment, as well as the single-center setting. Also, organ failure assessment was exclusively based on biomarkers and no functional tests such as echocardiography or extended invasive hemodynamic monitoring were integrated in analysis. Moreover, while the controls were sex-matched, age distribution is unfortunately not matched due to our method of acquisition of controls (healthy blood donors). It should also be noted that although the chosen cut-off of MRproADM showed good sensitivity, the clinical applicability of this cut-off might be limited by an overlap between the ranges in both survivors and nonsurvivors (Table 4).

At admission to the ICU, outcome prediction is of major interest. Identification and evaluation of novel biomarkers could improve current prognostic models (e.g., APACHE II, SOFA). In our study, we demonstrated the prognostic value of MRproADM in critical illness as a biomarker for ICU and overall mortality during a 26-month follow-up period. Future studies should aim at evaluating MRproADM as potential biomarker for guiding shock therapy and further validate its clinical usability, cost efficiency, and reliability in comparison to clinically established biomarkers.

## 5. Conclusions

Critically ill patients, especially with sepsis, display significantly increased levels of MRproADM. Its serum concentrations are closely associated with circulatory, renal, cardiac, and hepatic dysfunction. Elevated levels of MRproADM (cut − off > 1.4 nmol/L) are associated with ICU and overall mortality. Future studies should focus on validating its clinical usability and reliability as well as evaluating its possible use as a biomarker for guiding circulatory support.

## Figures and Tables

**Table 1:** Disease etiology of the study population.

	Sepsis	Nonsepsis
	*n* = 136	*n* = 67
Etiology of sepsis critical illness Site of infection, *n* (%)		
Pulmonary	71 (52.2%)	
Abdominal	26 (19.1%)	
Urogenital	8 (5.9%)	
Other	31 (22.8%)	
Etiology of nonsepsis critical illness, *n* (%)		
Cardiopulmonary disorder		28 (41.8%)
Acute pancreatitis		9 (13.4%)
Decompensated liver cirrhosis		8 (11.9%)
Severe gastrointestinal hemorrhage		4 (6%)
Nonsepsis other		18 (26.9%)

**Table 2:** Baseline patient characteristics and MRproADM serum measurements.

Parameter	All patients	Sepsis	Nonsepsis	*p*
Number	203	136	67	
Female, *n* (%)	79 (38.9)	56 (41.2)	23 (34.3)	n.s.
Age median, (range) (years)	64 (18-90)	65 (20-90)	62 (18-85)	n.s.
Charlson comorbidity index	2 (0-9)	2 (0-6)	2 (0-9)	n.s.
APACHE II score, median (range)	18 (2-43)	19 (4-43)	14 (2-33)	0.002
SOFA score, median (range)	9 (0-17)	9 (2-17)	8 (0-17)	n.s.
Mechanical ventilation, *n* (%)	137 (68.2)	91 (67.9)	46 (68.7)	n.s.
Vasopressor demand, *n* (%)	125 (61.5)	92 (67.6)	33 (49.3)	0.011
ICU days, median (range)	7 (1-137)	9 (1-137)	6 (1-45)	0.009
Death in ICU, *n* (%)	43 (21.2)	34 (25)	9 (13.4)	0.041
Overall mortality, *n* (%)	79 (41.1)	58 (45)	21 (33.3)	n.s.
MRproADM day 1, median (range) (nmol/L)	1.48 (0.1-35.2)	3.2 (0.1-35.2)	1.1 (0.1-8.7)	<0.001

For quantitative variables, median and range (in parenthesis) are given. Abbreviations: APACHE: acute physiology and chronic health evaluation; ICU: intensive care unit; MRproADM: midregional proadrenomedullin; SOFA: sequential organ failure assessment. ^∗^Significance between sepsis and nonsepsis patients was assessed using the Mann–Whitney *U* test, Fisher's exact test, or chi-squared test, respectively.

**Table 3:** Correlations of MRproADM with clinical scores and biomarkers of inflammation, organ failure and endothelial dysfunction, Spearman rank correlation test.

	All patients		Sepsis		Nonsepsis	
	*r*	*p*	*r*	*p*	*r*	*p*
Markers of inflammation						
CRP	0.487	<0.001	0.386	<0.001	0.361	0.003
Procalcitonin	0.550	<0.001	0.458	0.001	0.683	0.005
IL10	0.277	0.007	0.426	0.001	-0.054	n.s.
IL6	0.301	<0.001	0.190	n.s.	0.118	n.s.
TNF*α*	0.576	<0.001	0.539	0.008	0.658	0.02
Markers of organ dysfunction						
Creatinine	0.628	<0.001	0.644	<0.001	0.500	<0.001
Cystatin C	0.757	<0.001	0.702	<0.001	0.688	<0.001
AST	0.002	n.s.	-0.013	n.s.	0.158	n.s.
GLDH	-0.058	n.s.	0.006	0.013	-0.061	n.s.
Bilirubin total	0.040	0.016	-0.454	n.s.	0.321	0.008
*γ*GT	0.200	n.s.	-0.228	0.006	0.051	n.s.
PCHE	-0.499	<0.001	-0.506	<0.001	-0.515	<0.001
Prothrombin time	-0.272	<0.001	0.641	0.009	-0.408	n.s.
Albumin	-0.456	<0.001	0.644	<0.001	-0.273	n.s.
Urea	0.671	<0.001	0.702	<0.001	0.627	<0.001
Lactate	0.005	n.s.	0.177	0.042	-0.207	0.043
LDH	0.152	0.031	0.077	n.s.	0.248	0.001
NT-proBNP	0.602	<0.001	0.576	<0.001	0.445	0.0013
Fibrinogen	0.201	0.023	0.163	n.s.	0.064	n.s.
Markers of endothelial dysfunction						
ADMA	0.346	<0.001	0.327	<0.001	0.424	<0.001
SDMA	0.616	<0.001	0.591	<0.001	0.609	<0.001
CTproET1	0.870	<0.001	0.812	<0.001	0.853	<0.001
Clinical scores						
APACHE II	0.333	<0.001	0.242	0.007	0.316	0.014
SOFA	0.266	0.004	0.128	n.s.	0.390	0.014
SAPS2	0.460	<0.001	0.643	<0.001	0.248	n.s.

Abbreviations: *γ*GT: gamma-glutamyl transpeptidase; ADMA: asymmetric dimethylarginine; APACHE: acute physiology and chronic health evaluation score; AST: aspartate aminotransferase; CRP: C-reactive protein; CTproET1: C-terminal proendothelin-1; GLDH: glutamate dehydrogenase; IL10: interleukin 10; IL6: interleukin 6; LDH: lactate dehydrogenase; MRproADM: midregional proadrenomedullin; NT-proBNP: N-terminal pro-B-type natriuretic peptide; PCHE: pseudocholinesterase; SAPS2: simplified acute physiology score; SDMA: symmetric dimethylarginine; SOFA: sepsis-related organ failure assessment score; TNF*α*: tumor necrosis factor-*α*.

**Table 4:** Patient characteristics and comparison between survivors and nonsurvivors (ICU and overall survival).

Parameter	All patients	Survivor ICU	Nonsurvivor ICU	*p*	Survivors overall	Nonsurvivor overall	*p*
Number	203	160	43		113	79	
Female, *n* (%)	79 (38.9)	60 (37.5)	19 (44.2)	0.425	45 (39.8)	28 (35.4)	0.538
Age median, (range) (years)	64 (18-90)	62 (18-90)	71 (35-89)	0.002	60 (18-82)	69 (22-90)	<0.001
Charlson comorbitity index	2 (0-9)	2 (0-8)	3 (3-9)	0.001	2 (0-6)	3 (3-9)	<0.001
APACHE II score, median (range)	18 (2-43)	16 (2-40)	23 (5-43)	0.001	16 (3-40)	20 (5-43)	0.004
SOFA score, median (range)	9 (0-17)	7 (0-17)	12 (7-17)	<0.001	7 (0-17)	10 (3-17)	0.997
Mechanical ventilation, *n* (%)	137 (68.2)	96 (60.8)	41 (95.3)	<0.001	68 (60.2)	62 (79.5)	0.005
Vasopressor demand, *n* (%)	125 (61.5)	88 (55.0)	37 (86.0)	<0.001	60 (53.1)	58 (73.4)	0.004
ICU days, median (range)	7 (1-137)	7 (1-137)	7 (1-56)	<0.001	7 (1-137)	7 (1-66)	<0.001
Cystatin C, median (range) (mg/L)	1.5 (0.5-8.4)	1.4 (0.4-8.4)	1.9 (1.0-3.4)	0.182	1.3 (0.4-4.7)	1.7 (0.8-8.4)	0.073
CRP, median (range) (mg/dL)	103.5 (0-230)	92 (5-230)	118 (0-230)	0.158	84.5 (5-230)	121 (0-230)	0.064
Prothrombin time, median (range) (%)	70 (0-100)	71.3 (8-100)	69 (0-99)	0.170	74 (8-100)	69 (0-100)	0.110
Lactate, median (range) (mmol/L)	1.4 (0-19)	1.5 (0.5-10.3)	1.6 (0-19)	0.071	1.4 (0.4-10.3)	1.5 (0-19)	0.093
MRproADM day 1, median (range) (nmol/L)	1.48 (0.1-35.2)	2.2 (0.1-25.2)	3.6 (0.2-27.0)	0.017	2 (19.4-0.1)	3.2 (0.2-35.2)	0.006

For quantitative variables, median and range (in parenthesis) are given. Abbreviations: APACHE: acute physiology and chronic health evaluation; CRP: C-reactive protein; ICU: intensive care unit; MRproADM: midregional proadrenomedullin; SOFA: sequential organ failure assessment. ^∗^Significance between sepsis and nonsepsis patients was assessed using the Mann–Whitney *U* test, Fisher's exact test, or chi-squared test, respectively.

**Table 5:** Uni- and multivariate logistic regression analyses for MRproADM levels at ICU admission to predict ICU and overall mortality.

Parameter	ICU mortality				Overall mortality			
	Unadjusted OR (95% CI)	*p*	Adjusted OR (95% CI)	*p*	Unadjusted OR (95% CI)	*p*	Adjusted OR (95% CI)	*p*
MRproADM > 1.4 nmol/L	3.7 (1.47-9.29)	0.005	3.15 (1.08-9.20)	0.036	2.8 (1.44-5.55)	0.003	2.4 (1.12-5.34)	0.026
Lactate	1.159 (1.01-1.33)	0.040	1.20 (1.01-144)	0.043	1.19 (1.02-1.39)	0.027	1.25 (1.04-1-51)	0.016
Age	1.04 (1.01-1.06)	0.004	1.03 (0.99-1-06)	n.s.	1.04 (1.01-1.06)	0.001	1.03 (1.00-1-05)	n.s.
APACHE II	1.09 (1.04-1.14)	<0.001	1.06 (1.01-1.12)	0.014	1.06 (1.02-1.10)	0.004	1.03 (0.99-1-08)	n.s.

Abbreviations: 95% CI: 95% confidence interval; APACHE II: acute physiology and chronic health evaluation; MRproADM: midregional proadrenomedullin; OR: odds ratio.

**Table 6:** MRproADM performance in predicting ICU or overall mortality using a cut-off of 1.4 nmol/L.

	ICU mortality	Overall mortality
Sensitivity	86.1%	81.1%
Specificity	37.5%	39.8%
Positive predictive value	27.0%	48.5%
Negative predictive value	90.91%	75.0%
Positive likelihood ratio	1.38	1.35
Negative likelihood ratio	0.37	0.48

## Data Availability

Data is available upon request.

## Additional Points

*Key Findings.* MRproADM serum concentrations are significantly increased in critically ill patients compared to healthy controls. MRproADM serum concentrations are significantly elevated in patients with sepsis compared to other etiologies of critical illness. The diagnostic value of MRproADM for identifying sepsis is numerically lower than for established markers (CRP, PCT). MRproADM levels are correlated to organ dysfunction, inflammation, and clinical scores. MRproADM concentrations correlate with vasopressor use but not fluid balance. MRproADM levels are linked to ICU and overall survival during follow-up (up to 26 months).

## References

[1] Kitamura K., Kangawa K., Kawamoto M. (1993). Adrenomedullin: a novel hypotensive peptide isolated from human pheochromocytoma. *Biochemical and Biophysical Research Communications*.

[2] Cameron V. A., Fleming A. M. (1998). Novel sites of adrenomedullin gene expression in mouse and rat tissues. *Endocrinology*.

[3] Brain S. D., Grant A. D. (2004). Vascular actions of calcitonin gene-related peptide and adrenomedullin. *Physiological Reviews*.

[4] Kitamura K., Kangawa K., Eto T. (2002). Adrenomedullin and PAMP: discovery, structures, and cardiovascular functions. *Microscopy Research and Technique*.

[5] Eguchi S., Hirata Y., Iwasaki H. (1994). Structure-activity relationship of adrenomedullin, a novel vasodilatory peptide, in cultured rat vascular smooth muscle cells. *Endocrinology*.

[6] Shimekake Y., Nagata K., Ohta S. (1995). Adrenomedullin stimulates two signal transduction pathways, cAMP accumulation and Ca2+ mobilization, in bovine aortic endothelial cells. *The Journal of Biological Chemistry*.

[7] Hattori Y., Nakanishi N., Gross S. S., Kasai K. (1999). Adrenomedullin augments nitric oxide and tetrahydrobioptein synthesis in cytokine-stimulated vascular smooth muscle cells. *Cardiovascular Research*.

[8] Koo D. J., Zhou M., Chaudry I. H., Wang P. (2001). The role of adrenomedullin in producing differential hemodynamic responses during sepsis. *The Journal of Surgical Research*.

[9] Struck J., Tao C., Morgenthaler N. G., Bergmann A. (2004). Identification of an adrenomedullin precursor fragment in plasma of sepsis patients. *Peptides*.

[10] Yuyun M. F., Narayan H. K., Quinn P. A. (2017). Prognostic value of human mature adrenomedullin in patients with acute myocardial infarction. *Journal of Cardiovascular Medicine (Hagerstown, Md.)*.

[11] Legramante J. M., Mastropasqua M., Susi B. (2017). Prognostic performance of MR-pro-adrenomedullin in patients with community acquired pneumonia in the Emergency Department compared to clinical severity scores PSI and CURB. *PLoS One*.

[12] Liu D., Xie L., Zhao H., Liu X., Cao J. (2016). Prognostic value of mid-regional pro-adrenomedullin (MR-proADM) in patients with community-acquired pneumonia: a systematic review and meta-analysis. *BMC Infectious Diseases*.

[13] Pereira J. M., Azevedo A., Basilio C., Sousa-Dias C., Mergulhao P., Paiva J. A. (2016). Mid-regional proadrenomedullin: an early marker of response in critically ill patients with severe community-acquired pneumonia?. *Revista Portuguesa de Pneumologia*.

[14] Travaglino F., De Berardinis B., Magrini L. (2012). Utility of Procalcitonin (PCT) and Mid regional pro-Adrenomedullin (MR-proADM) in risk stratification of critically ill febrile patients in Emergency Department (ED). A comparison with APACHE II score. *BMC Infectious Diseases*.

[15] Schuetz P., for the TRIAGE Study group, Hausfater P. (2015). Biomarkers from distinct biological pathways improve early risk stratification in medical emergency patients: the multinational, prospective, observational TRIAGE study. *Critical Care*.

[16] Charles P. E., Peju E., Dantec A. (2017). Mr-Proadm elevation upon ICU admission predicts the outcome of septic patients and is correlated with upcoming fluid overload. *Shock*.

[17] Christ-Crain M., Morgenthaler N. G., Stolz D. (2006). Pro-adrenomedullin to predict severity and outcome in community-acquired pneumonia [ISRCTN04176397]. *Critical Care*.

[18] Enguix-Armada A., Escobar-Conesa R., De La Torre A. G., De La Torre-Prados M. V. (2016). Usefulness of several biomarkers in the management of septic patients: C-reactive protein, procalcitonin, presepsin and mid-regional pro-adrenomedullin. *Clinical Chemistry and Laboratory Medicine*.

[19] Guignant C., Voirin N., Venet F., Lepape A., Monneret G. (2012). Persistent high level of circulating midregional-proadrenomedullin and increased risk of nosocomial infections after septic shock. *Journal of Trauma and Acute Care Surgery*.

[20] Andaluz-Ojeda D., Nguyen H. B., Meunier-Beillard N. (2017). Superior accuracy of mid-regional proadrenomedullin for mortality prediction in sepsis with varying levels of illness severity. *Annals of Intensive Care*.

[21] Al Shuaibi M., Bahu R. R., Chaftari A.-M. (2013). Pro-adrenomedullin as a novel biomarker for predicting infections and response to antimicrobials in febrile patients with hematologic malignancies. *Clinical Infectious Diseases*.

[22] Koch A., Yagmur E., Linka J. (2018). High Circulating Caspase-Cleaved Keratin 18 Fragments (M30) Indicate Short- Term Mortality in Critically Ill Patients. *Disease Markers*.

[23] Singer M., Deutschman C. S., Seymour C. W. (2016). The third international consensus definitions for sepsis and septic shock (Sepsis-3). *JAMA*.

[24] Charlson M. E., Pompei P., Ales K. L., MacKenzie C. R. (1987). A new method of classifying prognostic comorbidity in longitudinal studies: development and validation. *Journal of Chronic Diseases*.

[25] Caruhel P., Mazier C., Kunde J., Morgenthaler N. G., Darbouret B. (2009). Homogeneous time-resolved fluoroimmunoassay for the measurement of midregional proadrenomedullin in plasma on the fully automated system B.R.A.H.M.S KRYPTOR. *Clinical Biochemistry*.

[26] Kim J. H. (2019). Multicollinearity and misleading statistical results. *Korean Journal of Anesthesiology*.

[27] DeLong E. R., DeLong D. M., Clarke-Pearson D. L. (1988). Comparing the areas under two or more correlated receiver operating characteristic curves: a nonparametric approach. *Biometrics*.

[28] Koch A., Yagmur E., Hoss A. (2018). Clinical relevance of copeptin plasma levels as a biomarker of disease severity and mortality in critically ill patients. *Journal of Clinical Laboratory Analysis*.

[29] Buendgens L., Yagmur E., Bruensing J. (2017). C-terminal proendothelin-1 (CT-proET-1) is associated with organ failure and predicts mortality in critically ill patients. *Journal of Intensive Care*.

[30] Koch A., Weiskirchen R., Bruensing J. (2013). Regulation and prognostic relevance of symmetric dimethylarginine serum concentrations in critical illness and sepsis. *Mediators of Inflammation*.

[31] Koch A., Weiskirchen R., Kunze J. (2013). Elevated asymmetric dimethylarginine levels predict short- and long-term mortality risk in critically ill patients. *Journal of Critical Care*.

[32] Angeletti S., Dicuonzo G., Fioravanti M. (2015). Procalcitonin, MR-proadrenomedullin, and cytokines measurement in sepsis diagnosis: advantages from test combination. *Disease Markers*.

[33] Mearelli F., Barbati G., Casarsa C. (2020). The integration of qSOFA with clinical variables and serum biomarkers improves the prognostic value of qSOFA alone in patients with suspected or confirmed sepsis at ED admission. *Journal of Clinical Medicine*.

[34] Yuyun M. F., Narayan H. K., Ng L. L. (2015). Prognostic significance of adrenomedullin in patients with heart failure and with myocardial infarction. *The American Journal of Cardiology*.

[35] Marino R., Struck J., Maisel A. S., Magrini L., Bergmann A., Somma S. (2014). Plasma adrenomedullin is associated with short-term mortality and vasopressor requirement in patients admitted with sepsis. *Critical Care*.

[36] Saeed K., Wilson D. C., Bloos F. (2019). The early identification of disease progression in patients with suspected infection presenting to the emergency department: a multi-centre derivation and validation study. *Critical Care*.

[37] Elke G., the SepNet Critical Care Trials Group, Bloos F. (2018). The use of mid-regional proadrenomedullin to identify disease severity and treatment response to sepsis - a secondary analysis of a large randomised controlled trial. *Critical Care*.

